# Toleranz und Akzeptanz der intratympanalen Injektion

**DOI:** 10.1007/s00106-022-01202-9

**Published:** 2022-08-02

**Authors:** Guido Mühlmeier, Matthias Tisch

**Affiliations:** Klinik für Hals-Nasen-Ohren-Heilkunde, Kopf- und Halschirurgie, Bundeswehrkrankenhaus Ulm, Oberer Eselsberg 40, 89081 Ulm, Deutschland

**Keywords:** Steroide, Hörsturz, Schmerz, Schwindel, Zweitlinien-Therapie, Steroids, Sudden hearing loss, Pain, dizziness, Second-line therapy

## Abstract

**Hintergrund:**

Die intratympanale Gabe von Steroiden ist Bestandteil der Therapie von akuten Hörverlusten. Bislang existieren nur wenig Daten zur Akzeptanz und Toleranz aus Sicht der Patient*innen.

**Material und Methoden:**

Zu den gesundheitlichen Voraussetzungen und den Umstäden ihrer intratympanalen Therapie wurden 84 Patient*innen befragt.

**Ergebnisse:**

Es stellten sich 38 Frauen und 46 Männer im Alter von durchschnittlich 57,2 Jahren meist auf Empfehlung ihres HNO-Arztes zur Zweitlinientherapie vor und empfanden die Injektionen zu 3,6 % als schmerzhaft, 22,6 % unangenehm, 67,9 % erträglich und zu 41,7 % als schmerzfrei (Mehrfachnennungen möglich). Eine Weiterempfehlung gaben 77,4 %, eine fehlende Empfehlung resultierte aus mangelnder Wirkung und unangenehmer Empfindung.

**Schlussfolgerung:**

Der weit überwiegende Anteil der Behandelten beurteilt die intratympanale Therapie als erträglich und gibt eine Weiterempfehlung. Die enge Patientenführung ist ein entscheidender Therapiebaustein.

## Abstract (en)

**Background:**

Intratympanic steroids are a therapeutic component in sudden hearing loss related to cochlear affection. Little has been published on patients’ acceptance and tolerance to date.

**Materials and methods:**

A total of 84 patients were asked about health conditions and circumstances surrounding their intratympanic therapy.

**Results:**

The patients comprised 38 women and 46 men, aged 57.2 years on average, who had mostly been referred by their ENT physician for second-line treatment. Reported were injection pain by 3.6%, discomfort by 22.6%, tolerability by 67.9%, and painlessness by 41.7% (more than one answer possible). Whereas 77.4% recommended intratympanic treatment, reasons for no recommendation were lack of efficacy and an unpleasant sensation.

**Conclusion:**

The majority of patients rated intratympanic therapy as bearable and recommendable. Close patient guidance seems to be an important part of the treatment.

Die Behandlung des Hörsturzes ist seit jeher Thema intensiver Diskussionen, da die Ursachen der Erkrankung zwar mit der Cochlea in Verbindung zu bringen sind, jedoch als multifaktoriell angenommen werden müssen. Gemäß der sich aktuell in Überarbeitung befindlichen Leitlinie Leitlinie Hörsturz (017-010) der Arbeitsgemeinschaft der wissenschaftlichen medizinischen Fachgesellschaften (AWMF) [1] ist ein Behandlungsversuch als Eilfall gerechtfertigt. Primär empfohlen werden hochdosierte, systemische Glukokortikoidtherapien, bei Kontraindikation oder nicht zufriedenstellender Wirkung intratympanale Behandlungen.

Seit mehr als einem Jahrzehnt finden die lokalen, intratympanalen Injektionen im Behandlungsalltag in deutschen Kliniken und Praxen Verbreitung und werden mittlerweile flächendeckend angeboten [2]. Als wirksame Bestandteile werden meist Fertigarzneimittel mit Dexamethasonphosphat (DM) oder Methylprednisolonsuccinat (MP) verwendet. Zur Erhöhung der Permeation und der Standzeit im Mittelohr wird in einigen Behandlungseinrichtungen der Injektionslösung Hyaluronsäure beigemischt [3].

Ein Therapiezyklus besteht im Regelfall aus drei Injektionen in einem Zeitraum von maximal 10 Tagen. Für die Patient*innen bedeutet dies eine Therapiezeit von jeweils etwa einer Stunde mit Lokalanästhesie des Trommelfells, der Injektion selbst und der Einwirkzeit nach erfolgter Injektion [4].

Für viele Patient*innen ist die Injektion „in das Ohr“ mit Angst und Zurückhaltung verbunden, die sich oft durch ein ausführliches Aufklärungsgespräch über Abläufe, Risiken, Chancen und Alternativen überwinden lässt. In der Literatur finden sich zahlreiche Artikel zu Methoden, Behandlungserfolgen und Komplikationen, eine Erhebung über Akzeptanz, Toleranz und Dyskomfort existiert nach unserer Recherche nur ansatzweise [5–7].

## Material und Methoden

In einer fortlaufenden Serie von 100 wegen eines Hörsturzes [1] intratympanal behandelten Patient*innen wurden im Rahmen der Zertifizierung der Hals-Nasen-Ohren-Klinik am Bundeswehrkrankenhaus Ulm Befragungen zu den Umständen der Behandlung durchgeführt. Der Rücklauf vollständig ausgefüllter und verwertbarer Bögen lag bei 84 %. Das auswertbare Patient*innenkollektiv bestand aus 38 Frauen (45,2 %) und 46 Männern (54,8 %) mit einem Durchschnittsalter von 57,2 (±14,9) Jahren (Tab. 1).

**Table 1:** 

	Alter (Jahre)	Abstand Hörsturz – ITI (Tage)	Vorerkrankungen^*^ (%)	Rate an Rezidivhörstürzen (%)	Begleitschwindel (%)	Begleittinnitus (%)
W (*n* = 38)	54,2 ± 15,5	21 ± 9,2	21,1	21,1	23,7	76,3
M (*n* = 46)	59,7 ± 14,0	24,5 ± 11,3	47,8	30,4	17,4	78,3

^* ^signifikanter Unterschied (*p* < 0,005)

Vorausgegangen war bei allen Patient*innen eine leitliniengerechte Therapie, im Regelfall mit hochdosierten systemischen Steroiden außer bei Vorliegen von Kontraindikationen. Im Bundeswehrkrankenhaus Ulm erfolgte dann eine Serie von mindestens drei intratympanalen Injektionen durch das Trommelfell aufgrund eines reintonaudiometrisch verifizierten Hörsturzes. Die einzelne Behandlung startete mit einer Ohrmikroskopie zur Feststellung eines reizlosen Zustands des Gehörgangs und eines geschlossenen und zur intratympanalen Behandlung geeigneten Trommelfells des betroffenen Ohrs. Die Lokalanästhesie wurde mit handelsüblicher Lidocain-Sprühlösung über ca. 10 min durchgeführt. Vor Beginn der Injektion wurden jeweils 1,0 ml DM 4 mg/ml (Ampulle als Fertigarzneimittel) und 0,5 ml Hyaluronsäure (EDO) vermischt und von dieser Lösung ca. 0,7 ml in eine 1 ml fassende Spritze aufgezogen. Die Injektion der durch die Patient*innen für die Zeitdauer der Lokalanästhesie in der Hand vorgewärmten Lösung erfolgte unter mikroskopischer Sicht mit einer Injektionsnadel 0,5 × 60 mm mit vorderer Verjüngung auf 0,3 mm in den zentralen Anteil des Trommelfells dorsal des Umbo membranae tympani mit einer Injektionsmenge von ca. 0,4 ml. Nach der Injektion lagen die Patient*innen für ca. 20 min auf der gegenüberliegenden Seite. Tonaudiometrische Untersuchungen wurden vor jeder Injektion und ca. 2 Wochen nach Abschluss der Therapie durchgeführt.

Die Befragung zur intratympanalen Behandlung mittels eines Rasters erfolgte ca. 2 Wochen nach Abschluss der Therapie im Rahmen der Nachuntersuchung. Erhoben wurden Alter und Geschlecht, die Dauer nach Hörsturzereignis bis zur ersten intratympanalen Injektion, die Vorbehandlung mit oralen Steroiden, intravenösen Steroiden und weiteren Medikamenten, Vorerkrankungen (arterieller Hypertonus, Herzerkrankungen, Lebererkrankungen, Nierenerkrankungen, Diabetes), die Überweisung durch einen HNO-Facharzt, die Symptomatik des Hörsturzes mit Schwindel, Tinnitus und einem Rezidivereignis sowie die Umstände der Injektionen (Schwindel bei Injektion, schmerzhaft, unangenehm, erträglich, schmerzfrei; Tab. 2) und schließlich die Frage nach einer Weiterempfehlung der Therapie (Tab. 3). Die Studie wurde durch die Ethik-Kommission der Universität Ulm mit Votum vom 24.06.2021 positiv bewertet (259/21).

**Table 2:** 

	Schmerzhaft (%)	Unangenehm (%)	Erträglich (%)	Schmerzfrei (%)	Weiterempfehlung (%)
Alle (*n* = 84)	3,6	22,6	67,9	41,7	77,4
W (*n* = 38)	5,3	26,3	60,5	42,1	73,7
M (*n* = 46)	2,2	19,6	73,9	41,3	80,4

**Table 3:** 

	Vorausgehende Therapie^*^				Wahrnehmung der ITI			
Empfehlung	Steroide oral (%)	Steroide i.v. (%)	Andere	Rezidivhörsturz (%)	Schmerzhaft^**^ (%)	Unangenehm (%)	Erträglich (%)	Schmerzfrei (%)
Ja (*n* = 65)	30,8	50,8	21,5	29,2	1,5	24,6	67,7	43,1
Nein (*n* = 19)	47,4	57,9	47,4	15,8	10,5	15,8	68,4	36,8

^*^ signifikanter Unterschied zwischen Empfehlenden und nicht Empfehlenden hinsichtlich der Anzahl vorausgehender Therapien (*p* < 0,012)

^** ^signifikanter Unterschied (*p* < 0,03)

## Ergebnisse

84 vollständig ausgefüllte Befragungsbögen standen zur Auswertung zur Verfügung. Diese wurden von 38 Frauen (45,2 %) und 46 Männern (54,8 %) im Alter von 57,2 Jahren (±14,9, Spannbreite: 29 bis 90 Jahre) ausgefüllt. Bei 26,2 % handelte es sich um ein Rezidivereignis, was bei Männern mit 30,4 % häufiger auftrat als bei Frauen (21,1 %; *p* = 0,17). Im Median begann die intratympanale Therapie 21 (95%-Konfidenzintervall: 15) Tage nach Auftreten des Hörsturzes.

79,8 % wurden vom HNO-Arzt überwiesen. Ein kleinere Gruppe von Patient*innen stellte sich primär vor (10,7 %) oder wurde vom Facharzt für Allgemeinmedizin oder Neurologie (9,5 %) empfohlen.

21,4 % erhielten eine primäre intratympanale Therapie wegen Kontraindikationen in Bezug auf systemische Steroide. Vorerkrankungen lagen bei 35,7 % vor, teilweise mit mehrfachen Angaben, signifikant häufiger bei Männern (*p* < 0,005).

In Verbindung mit dem ersten Auftreten des plötzlichen Hörverlusts berichteten 20,2 % über vorübergehenden Schwindel und 77,4 % über begleitenden Tinnitus. Zwischen den Vergleichsgruppen nach Geschlecht und Weiterempfehlung der Therapie ergaben sich keine signifikanten Unterschiede.

Über die Nebenwirkungen der Injektion gaben 14,3 % ein leichtes Schwindelgefühl an, das nach spätestens 2 min vollständig verschwunden war. 3,6 % beurteilten die Injektion als schmerzhaft, 22,6 % als unangenehm und 41,7 % als schmerzfrei. 67,9 % empfanden die Injektion als erträglich. Insgesamt beurteilten 89,3 % die Injektion als erträglich und/oder schmerzfrei (*p* < 0,0001). Diese Patient*innen wiesen gegenüber der Gesamtgruppe eine fast gleiche Verteilung der Auswerteparameter auf. Ein nicht signifikanter Trend bestand aufseiten der Frauen, die gegenüber Männern die Injektionen als schmerzhafter und unangenehmer einstuften.

Eine Weiterempfehlung gaben 77,4 % der Patient*innen, 80,4 % der Männer und 73,7 % der Frauen. Wurde die Therapie nicht weiterempfohlen (22,6 %), waren bei 20,2 % die mangelnde Wirkung und bei 2,4 % der Dyskomfort während der Behandlung die Gründe für die Ablehnung. Bei einer Weiterempfehlung beurteilten 1,5 % die Behandlung als schmerzhaft im Vergleich zu 10,5 % der Ablehnenden (*p* < 0,03) und wiesen mit 29,2 % gegenüber 15,8 % häufiger vorausgehende Hörsturzereignisse auf (*p* = 0,12). Die mediane Dauer vom Hörsturz bis zum Beginn der intratympanalen Therapie lag bei Weiterempfehlenden bei 14 Tagen im Vergleich zu 42 Tagen bei Ablehnenden (*p* < 0,02).

## Diskussion

Die intratympanale Therapie findet nach fast zwei Jahrzehnten in Deutschland sowohl in Kliniken als auch in Praxen eine flächendeckende Anwendung [2]. Entsprechend der Leitlinie [1] wird diese in den weitaus meisten Fällen als Zweitlinientherapie bei nicht zufriedenstellenden Ergebnissen der systemischen Glukokortikoidtherapie angewendet.

Die von uns behandelten Patient*innen wiesen eine Geschlechtsverteilung und ein Altersspektrum auf, wie es in der Mehrzahl der publizierten größeren Studien beschrieben wurde [8–10]. Begleitender Schwindel wird in der Literatur mit 29–56 % Häufigkeit angegeben und stellt einen prognostisch ungünstigen Faktor für die Erholung des Hörverlusts dar [11]. Mit 20,2 % lagen unsere Patient*innen unterhalb dieser Werte, was durchschnittlich für leichtere Hörsturzereignisse spricht. Die Rate an begleitendem Tinnitus lag hingegen mit 77,4 % in der Mitte der Literaturangaben, die Werte zwischen 41 und 90 % beschreiben [11, 12]. Die durchgehenden Ohrgeräusche verursachen bei vielen Betroffenen sekundäre psychosomatische Reaktionen und erschweren den Leidensdruck durch den Hörverlust im Rahmen des Hörsturzes [13].

In der Literatur werden zahlreiche Daten zu Erfolgen der intratympanalen Therapie in Abhängigkeit vom verwendeten Wirkstoff, der Dauer nach Ereignis und dem Schweregrad des Hörverlusts publiziert (ca. 50 pro Jahr) und in Reviews zusammengefasst [14]. Lediglich zwei Arbeiten [6, 7] machen Angaben zu Schmerzen durch die Injektion, eine von Demirhan et al. [6] zu einem VAS-Score bezüglich Schmerzen 5 und 60 min nach Injektion. Diese Angaben beziehen sich auf den Vergleich vom besser verträglichen DM zum in dieser Publikation besser wirksamen MP, das jedoch einen für das Mittelohr ungünstigeren pH-Wert aufweist und daher ein stärkeres Brennen nach Injektion hervorruft.

Bei einem VAS-Score von 4,3 zu einem Zeitpunkt von 5 min nach Injektion von DM ohne Lokalanästhesie [6] lässt sich nur ein indirekter Vergleich mit unseren Daten durchführen. Ausgehend von einer mit 41,7 % hohen Rate an schmerzfreien und 67,9 % als erträglich einstufenden Patient*innen beurteilten 89,3 % die Injektion als tolerabel, was im Verhältnis zu den Daten von Demirhan positiver ausfällt. Im Vergleich zu einer Rate von 47,2 % an Schmerzen im Rahmen der Injektion mit einem VAS von 3,2 bei Hu et al. [7] liegt die bei unseren Patient*innen erhobene Rate deutlich niedriger, was auch mit der Betreuung der Patient*innen während der Injektion zusammenhängen könnte. Gleichzeitig kann angenommen werden, dass die Beimengung von Hyaluronsäure zu einer Pufferung der Lösung führt, die die Reaktion im Mittelohr für die Patient*innen angenehmer macht.

Die niedrige Rate von 14,3 % an kurzzeitigem Schwindel nach Injektion hängt mit dem Vorwärmen der Injektionslösung in der Hand oder unter der Achsel während der Einwirkzeit des Lokalanästhetikums zusammen. Handwärme liegt bei 34–35 °C, Achselwärme bei 36–37 °C. Diese Temperaturen führen zu einer geringen thermischen Belastung des Innenohrs, die bei empfindlicher Wahrnehmung in Einzelfällen Schwindel auslösen kann. Ata et al. [15] wiesen signifikante Unterschiede in der Schwindelhäufigkeit zwischen zimmerwarmer und körperwarmer Injektionslösung nach und beschrieben ein rasches Abklingen innerhalb weniger Minuten. Im Vergleich zu einer technischen Erwärmung der Injektionslösung auf Körpertemperatur stellt die beschriebene Methode ein pragmatisches, zielführendes und wenig aufwendiges Verfahren dar.

Der Vergleich der Anzahl der Vorbehandlungen vor der intratympanalen Therapie lag bei Patient*innen ohne Weiterempfehlung signifikant höher als mit Weiterempfehlung (*p* < 0,012). Hierin spiegelt sich die psychosomatische Seite des Hörsturzes wider und kennzeichnet die Belastungssituation der Betroffenen, vergleichbar auch beschrieben von Lamparter et al. [16]. Schüssler et al. [15] sahen bei ihren Patient*innen psychosoziale Faktoren als wichtig für die Auslösung eines Hörsturzes an, auch wenn sich keine konkreten Persönlichkeitsmuster herausarbeiten ließen. Angstbehaftete Patient*innen neigten häufiger dazu, viele verfügbare Therapiemethoden auszuprobieren, um keine wirksame zu verpassen [17].

Im Zusammenhang mit der Aufklärung geben die meisten Patient*innen Angst oder Unbehagen gegenüber der intratympanalen Behandlung an. (Hierbei handelt es sich um eine langjährige Beobachtung über mehrere Tausend Patient*innen hinweg.) Das Attestieren einer positiven Bewertung nach erfolgter Therapie hängt mit der Art der Aufklärung und noch wesentlicher mit der Patientenführung während der Erstinjektion zusammen. Werden die Patient*innen über die zu erfolgenden Einzelschritte rechtzeitig informiert, können sie sich darauf vorbereiten und empfinden dies entspannter, weil negative Überraschungen ausbleiben. Wichtig in diesem Zusammenhang ist der Hinweis auf ein gedämpftes Hören für die Zeit des Flüssigkeitsverbleibs im Mittelohr, die sich abhängig vom Entleeren über die Perforation im Rahmen der Injektion oder die Tuba Eustachii bis zu 2 h hinziehen kann. In dieser Zeit wird auch der begleitende Tinnitus als wesentlich intensiver wahrgenommen.

Zusammenfassend stellt die intratympanale Therapie eine komplikationsarme, von Patient*innen gut tolerierte und überwiegend sehr gut akzeptierte Behandlungsmethode dar.

## Fazit für die Praxis

- Die intratympanale Therapie hat ihren festen Stellenwert bei akuten Hörverlusten durch Affektion der Cochlea eingenommen.
- Für die Patient*innen ist allein die Vorstellung der Injektionen mit Angst und Unbehagen begleitet, weshalb eine empathische Aufklärung gleichsam wie eine erklärende Begleitung bei den Injektionen wichtig ist.
- Zur Reduktion von unangenehmen Begleiterscheinungen der Injektion sind die Art der Betäubung des Trommelfells, das Anwärmen der Lösung und die Entlüftung der Pauke während der Injektion von Bedeutung.
